# The Association Between Genetic Variation in *FOXP2* and Sensorimotor Control of Speech Production

**DOI:** 10.3389/fnins.2018.00666

**Published:** 2018-09-20

**Authors:** Siyun Zhang, Jiangli Zhao, Zhiqiang Guo, Jeffery A. Jones, Peng Liu, Hanjun Liu

**Affiliations:** ^1^Department of Rehabilitation Medicine, The First Affiliated Hospital, Sun Yat-sen University, Guangzhou, China; ^2^Department of Computer Science and Technology, Zhuhai College of Jilin University, Zhuhai, China; ^3^Department of Psychology, Laurier Centre for Cognitive Neuroscience, Wilfrid Laurier University, Waterloo, ON, Canada; ^4^Guangdong Provincial Key Laboratory of Brain Function and Disease, Zhongshan School of Medicine, Sun Yat-sen University, Guangzhou, China

**Keywords:** auditory feedback, sensorimotor integration, *FOXP2*, genetic variant, event-related potential

## Abstract

Significant advances have been made in understanding the role of auditory feedback in sensorimotor integration for speech production. The neurogenetic basis of this feedback-based control process, however, remains largely unknown. Mutations of *FOXP2* gene in humans are associated with severe deficits in speech motor behavior. The present study examined the associations between a *FOXP2* common variant, rs6980093 (A/G), and the behavioral and event-related potential (ERP) responses to -50 and -200 cents pitch perturbations during vocal production in a sample of 133 Chinese adults. Behaviorally, the GG genotype was associated with significantly smaller vocal compensations for -200 cents perturbations relative to the AA and AG genotypes. Furthermore, both the AA and AG genotypes exhibited significant positive correlations between the degree of vocal compensation for -50 and -200 cents perturbations and the variability of normal voice fundamental frequency, whereas no such correlation existed for the GG genotype. At the cortical level, significantly larger P2 responses to -200 cents perturbations were associated with the GG genotype as compared to the AA and AG genotypes due to increased left-lateralized activity in the superior, middle, and inferior frontal gyrus, precentral gyrus, anterior cingulate cortex, middle temporal gyrus, and insula. The neurobehavioral responses to -50 cents perturbations, however, did not vary as a function of genotype. These findings present the first neurobehavioral evidence for an association between *FOXP2* genetic variant and auditory-motor integration for vocal pitch regulation. The differential effects of *FOXP2* genotypes at rs6980093 may reflect their influences on the weighting of feedback and feedforward control of speech production.

## Introduction

Speech production relies on the integration of auditory feedback into the vocal motor system in the brain (Hickok et al., 2011). Although compensatory adjustments of vocal output in response to altered auditory feedback have been well documented (Burnett et al., 1998; Houde and Jordan, 1998; Macdonald et al., 2010), the neural mechanisms underlying auditory-motor integration are far from being understood. Event-related potentials (ERPs) of the N1-P2 complex have been identified in the cortical processing of pitch feedback perturbations during vocal production (Behroozmand and Larson, 2011; Liu H. et al., 2011; Scheerer et al., 2013), which are thought to, respectively, reflect the early pre-attentive detection of feedback errors and later cognitive processing of auditory-motor transformations. Neuroimaging studies have revealed a complex neural network in the fronto-temporo-parietal regions to be involved in auditory feedback control of speech production (Tourville et al., 2008; Zarate and Zatorre, 2008; Parkinson et al., 2012; Chang et al., 2013; Behroozmand et al., 2015, 2016). Changes to these critical regions caused by neurological diseases, such as Parkinson’s disease (PD), Alzheimer disease (AD), and temporal lobe epilepsy (TLE), lead to disorders of speech motor control as reflected by abnormal vocal compensations and/or associated brain activity (Liu et al., 2012; Huang et al., 2016; Li et al., 2016; Mollaei et al., 2016; Ranasinghe et al., 2017).

Some individuals, however, suffer from speech disorders that are inheritable, such as developmental verbal dyspraxia (MacDermot et al., 2005), stuttering (Ambrose et al., 1997), and phonological processing disorders related to 16p11.2 deletions (Hippolyte et al., 2016). These genetic speech disorders are associated with impaired sensorimotor processing of speech production. For example, individuals with developmental verbal dyspraxia have difficulties in controlling orofacial muscles (Watkins et al., 2002a), and individuals with stuttering and 16p11.2 deletions carriers have shown atypical compensations for speech feedback perturbations (Cai et al., 2012; Demopoulos et al., 2018). Despite significant progress in the identification of risk genes associated with speech and language disorders (Graham and Fisher, 2013; Konopka and Roberts, 2016), the neurogenetic basis of speech motor control remains largely unknown.

One milestone in the exploration of the link between genetics and speech and language disorders was the discovery of *FOXP2* gene mutations that disrupt the DNA-binding site of the protein in a landmark multigenerational study of the KE family (Lai et al., 2001). As a monogenic speech disorder caused by *FOXP2* mutations, developmental verbal dyspraxia is characterized by deficits in the production of orofacial motor sequences necessary for fluent speech (Hurst et al., 1990; Vargha-Khadem et al., 1998) and impaired grammatical skills (Watkins et al., 2002a). There is also evidence that suggests associations between *FOXP2* mutations and speech sound disorders (Zhao et al., 2010). The *FOXP2* gene encodes a forkhead domain transcription factor that is expressed in the cortico-striatal network, including the lateral frontal and temporo-parietal cortices, basal ganglia, and cerebellum (Ferland et al., 2003; Lai et al., 2003; Teramitsu et al., 2004). Abnormalities in gray matter density in this network have been identified in structural imaging studies on individuals with *FOXP2* mutations (Vargha-Khadem et al., 1998; Watkins et al., 2002b; Belton et al., 2003; Padovani et al., 2010; Premi et al., 2012). Functional imaging studies of overt speech production have shown decreased brain activity in Broca’s area and putamen in individuals with *FOXP2* mutations (Liegeois et al., 2003) and associations between *FOXP2* genotypes and variations of activation in the IFG in healthy populations (Pinel et al., 2012). Behaviorally, the impact of *FOXP2* gene on learning of auditory-motor interactions is reflected by associations between individual differences in speech category learning and variation in the *FOXP2* gene (Chandrasekaran et al., 2015). Also, animal studies have shown disruptions in vocal learning and vocal-motor variability in songbirds after knockdown of *FoxP2* (Haesler et al., 2007; Murugan et al., 2013) and adult mice with *Foxp2* mutation (Chabout et al., 2016). These studies in animals and humans have implicated a role of *FOXP2* in sensorimotor processing. On the other hand, activation of brain regions within the cortico-striatal network, such as the superior temporal gyrus (STG), inferior frontal gyrus (IFG), inferior parietal lobule (IPL), putamen, and thalamus, has been identified in the auditory-motor processing of feedback errors during speech production (Tourville et al., 2008; Zarate and Zatorre, 2008; Parkinson et al., 2012; Behroozmand et al., 2015; Tang et al., 2018). These findings led us to hypothesize an association between *FOXP2* and sensorimotor control of speech production. There is so far, however, no direct evidence to support this hypothesis.

To test this hypothesis, we correlated a *FOXP2* single nucleotide polymorphism (SNP rs6980093) that involves the adenine (A) and guanine (G) exchange with neurobehavioral responses to feedback errors during speech production. We identified rs6980093 as target SNP because of its associations with variations of brain activity in the IFG during speech-related reading tasks (Pinel et al., 2012), individual differences in language and reading skills (Mozzi et al., 2017), and speech category learning abilities (Chandrasekaran et al., 2015). Healthy young adults were instructed to produce sustained vowel sounds while they heard pitch perturbations in their voice auditory feedback. Compensatory vocal responses and ERP responses known as the N1-P2 complex were measured and compared as a function of *FOXP2* genotype. Given the important role of *FOXP2* in tuning the function of cortico-striatal networks that regulate critical aspects of speech, language, and motor control (Fisher and Scharff, 2009; Lieberman, 2009; Konopka and Roberts, 2016), we hypothesized that an association would exist between the *FOXP2* gene and auditory-vocal integration, as reflected by the modulation of vocal compensations and ERP responses by variation in SNP rs6980093.

## Materials and Methods

### Subject

One hundred and fifty college students were recruited from Sun Yat-sen University of China to participate in this experiment. All participants were right-handed and from the population of Han Chinese. The inclusion criteria were as follows: no prior history of neurological or psychiatric diseases, no speech, hearing or language disorders, non-smokers, and no history of taking neuroactive substances (e.g., alcohol, caffeine, drugs, etc.). Written informed consent was obtained from all participants, and the research protocol was approved by the Institution Review Board of The First Affiliated Hospital at Sun Yat-sen University of China in accordance with the Code of Ethics of the World Medical Association (Declaration of Helsinki).

### Genotyping

All participants were genotyped for the polymorphism in *FOXP2* (SNP rs6980093). DNA was extracted from saliva samples collected with the Oragene^TM^ DNA collection kit (OG-500, from DNA Genotek) following standard instructions. The DNA of all participants was genotyped for a polymorphism in SNP rs6980093. Sequencing was performed by the dideoxy-chain termination method (ABI Applied Biosystems) using an ABI 3730XI Real-Time PCR System.

The frequency of the *FOXP2* (SNP rs6980093) genotypes (*n* = 150; AA: *n* = 55; AG: *n* = 71; GG: *n* = 24) did not significantly deviate from the Hardy–Weinberg equilibrium (Chi-square test, χ^2^ = 0.018, *p* = 0.892). Of the 150 participants who eventually entered the study, 17 participants were excluded because their electroencephalography (EEG) data did not reach the criteria of inclusion (see *EEG data analyses*). Thus, the data from 133 young adults aged 18–29 years (mean age 21 ± 2 years) were collected in the present experiment. The participants were divided into three groups according to their genotypes: AA (*n* = 49; 14 males), AG (*n* = 63; 18 males), and GG (*n* = 21; 4 males). The three groups did not differ in their age [*F*(2,130) = 1.649, *p* = 0.196] and gender (χ^2^ = 0.333, *p* = 0.564).

### Procedure

All participants completed a vocal production experiment using the frequency altered feedback (FAF) paradigm after the collection of their saliva. They were cued to start and stop vocalizing when a blue indicator light on a computer screen was on and off, resulting in a stable vocalization that was 3 s in length. During each vocalization, participants heard their own voice unexpectedly pitch-shifted downward 50 or 200 cents (100 cents = 1 semitone) once. Each pitch shift had a fixed duration of 200 ms and occurred 1500–2000 ms after the onset of vocalization. The two sizes of pitch perturbations were pseudo-randomly presented to all participants. Prior to initiating next vocalization, participants were required to take a break of 2–3 s to avoid vocal fatigue. They produced 200 consecutive vocalizations that led to a total of 200 trials, including 100 trials for the -50 cents perturbations and 100 trials for the -200 cents perturbations.

### Apparatus

Participants were tested in a sound-treated booth. In order to partially mask their air-borne and bone-conducted feedback, the acoustical recording system was calibrated prior to data recording, ensuring that the intensity of voice feedback was 10 dB sound pressure level (SPL) higher than that of participant’s vocal output. The voice signals were transduced by a dynamic microphone (DM2200, Takstar Inc.) and sent to an Eventide Eclipse Harmonizer via a MOTU Ultralite Mk3 Firewire audio interface. A custom-developed MIDI software program (Max/MSP v.5.0 by Cycling 74) was used to control the Eventide Eclipse Harmonizer to pitch-shift the voice signals. Acoustical parameters, including the direction, magnitude, and inter-stimulus interval (ISI) of the pitch perturbations, were manipulated by this program. Also, this program was used to generate transistor-transistor logic (TTL) control pulses that marked the onset of the pitch perturbation. Finally, the pitch-shifted voice signals were amplified by an ICON NeoAmp headphone amplifier and fed back to participants through insert earphones (ER1-14A, Etymotic Research Inc.). The original and pitch-shifted voice signals as well as the TTL pulses were digitized with a sampling frequency of 10 kHz by a PowerLab A/D converter (model ML880, AD Instruments) and recorded using LabChart software (v.7.0 by AD Instruments).

The EEG signals were recorded from each participant’s scalp with a 64-electrode Geodesic Sensor Net (Electrical Geodesics Inc.). A high input-impedance Net Amps 300 amplifier (Z_in_ ≈ 200 MΩ; Electrical Geodesics Inc.) was used to amplify the EEG signals. This amplifier accepts scalp-electrode impedances up to 40–60 kΩ, thus the impedance levels of individual sensors were carefully adjusted to be less than 50 kΩ and maintained throughout the recording (Ferree et al., 2001). All channels were referenced to the vertex (Cz) during the online recording (Ferree et al., 2001). The TTL pulses that marked the onset of the pitch perturbation were sent to the EEG recording system via a DIN cable. Finally, the EEG signals were digitized at a sampling frequency of 1000 Hz and saved onto a Mac Pro computer using NetStation software (v.4.5, Electrical Geodesics Inc.).

### Vocal Responses Measurement

The magnitude and latencies of compensatory vocal responses to pitch-shifted auditory feedback were measured using IGOR PRO software (v.6.0, WaveMetrics Inc.). Voice fundamental frequency (*F*_0_) contours in Hertz were extracted from voice signals using Praat software (Boersma, 2001) and converted to the cent scale with the following formula: cents = 100 × [12 × log_2_(*F*_0_/reference)] [reference = 195.997 Hz (G3 note)]. We then segmented the voice *F*_0_ contours in cents into epochs using a window of -200 ms to 700 ms relative to the onset of the pitch perturbation and performed a waterfall procedure to visually inspect all individual trials. Following this procedure, bad trials that were contaminated by vocal interruptions or signal processing errors were excluded from further analyses. Those trials whose direction opposed the downward perturbations were defined as compensatory responses and entered the averaging procedure. Finally, 81% of compensatory vocal trials that contained no artifacts were normalized by subtracting the mean *F*_0_ values in the 200 ms baseline period from the *F*_0_ values after the perturbation onset and averaged to generate an overall compensatory response for each condition. The magnitude of a vocal response in cents was defined as the greatest *F*_0_ value following the response onset. The latency was measured as the peak time when the voice *F*_0_ contours reached a maximum value. Additionally, the SD of the baseline mean *F*_0_ for the averaged response was measured as an index of the variability of the participant’s voice in the absence of feedback perturbations.

### EEG Data Analysis

The EEG data were submitted to NetStation software for offline analysis. First, they were band-pass filtered using a filter with cut-off frequencies of 1–20 Hz. Following a segmentation procedure, the filtered EEG signals were segmented into 500 ms post-stimulus epochs with a 200 ms pre-stimulus baseline. Next, an artifact detection procedure was performed on the segmented epochs to reject trials with segments that included voltage values that exceeded ±55 μv of the moving average over an 80-ms window, which is typically the result of excessive muscular activity, eye blinks, or eye movements. Individual electrodes were rejected if they contained artifacts in more than 20% of the epochs, and individual files were excluded from the averaging procedure if they contained more than 10 bad channels. In order to ensure appropriate rejections of those trials with artifacts, we performed an additional visual inspection of all the individual trials. On average, 81% of individual trials were retained for averaging. Finally, artifact-free trials were re-referenced to the average of the electrodes on each mastoid, averaged, and baseline-corrected to generate an overall response for each condition. The amplitudes and latencies of the N1 and P2 components were measured from 10 electrodes (FC1, FC2, FC3, FC4, FCz, C1, C2, C3, C4, and Cz) as the negative and positive peaks in the time windows of 80–160 ms and 180–280 ms after the onset of pitch perturbation, respectively, since the N1 and P2 responses to pitch-shifted voice auditory feedback are primarily pronounced in the frontal and central areas (Hawco et al., 2009; Chen et al., 2012).

In order to further evaluate the effect of genetic variation on the neural networks that support the cortical processing of voice auditory feedback, we performed source localization of N1 and P2 responses across the genotypes using standard low-resolution electromagnetic tomography (sLORETA) (Pascual-Marqui, 2002). Previous fMRI and intra-cerebral recordings studies (Mulert et al., 2004; Zumsteg et al., 2006) have validated the sources estimated by sLORETA, and this method has been successfully applied to localizing the cortical generators of N1 and P2 responses to pitch-shifted voice auditory feedback (Huang et al., 2016; Guo et al., 2017). For sLORETA, the standardized current density was calculated with a dense grid of 6239 voxels at a spatial resolution of 5 mm in the Montreal Neurological Institute (MNI)-reference brain. The voxel-based sLORETA images were calculated in a realistic standardized head model that was computed with the boundary element (BEM) approach (Fuchs et al., 2002) using the MNI152 template (Mazziotta et al., 2001). In the present study, the voxel-based sLORETA images were computed at a 5 ms time window centered at the maximal global field power peaks within the N1 and P2 time windows, and compared across the genotypes using sLORETA-built-in-voxelwise randomization tests with 10000 permutations based on statistical non-parametric mapping (SnPM) for multiple comparison employing a log-*F*-ratio statistic. The voxels with significant differences (corrected *p* < 0.05) were specified in MNI coordinates and labeled as Brodmann areas (BA) within the EEGLAB software (Delorme and Makeig, 2004).

### Statistical Analysis

Repeated-measures analyses of variance (RM-ANOVAs) were conducted on the vocal and ERP responses (N1 and P2) to pitch perturbations in SPSS (v.20.0). The magnitudes and latencies of vocal compensations were subject to two-way RM-ANOVAs, including a within-subject factor of stimulus magnitude (-50 and -200 cents) and a between-subject factor of genotype (AA, AG, and GG). The magnitudes and latencies of N1 and P2 responses were analyzed using three-way RM-ANOVAs in which stimulus magnitude and electrode site were regarded as within-subject factors while genotype was regarded as a between-subject factor. Subsidiary RM-ANOVAs were conducted in the case of significant higher-order interactions between any of those variables. *Post hoc* analyses were performed using Bonferroni adjustment for multiple comparisons. Probability values were corrected for multiple degrees of freedom when the sphericity assumption was violated. The size of differences across the conditions was described by calculating effect size indexed by $\eta_{p}^{2}$. An alpha level of 0.05 was considered significant for all statistical analyses.

## Results

### Vocal Responses

**FIGURE 1:**
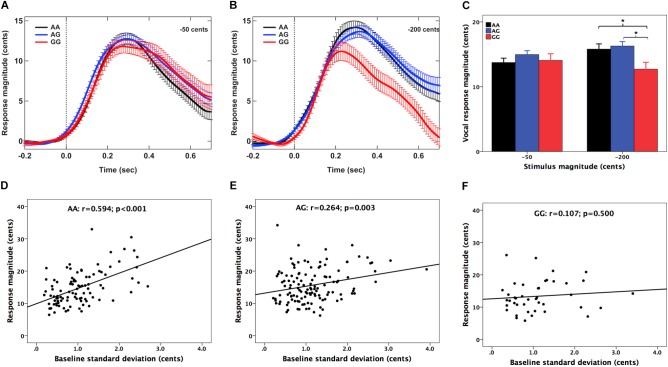
**(A,B)** Grand-averaged voice *F*_0_ contours in responses to –50 and –200 cents pitch perturbations for the AA (black), AG (blue), and GG (red) genotypes. Errors bars represent standard errors of the means (SEM). **(C)** T-bar plots of the magnitudes (mean + SEM) of vocal compensations across the genotypes, showing that the GG genotype exhibited significantly smaller magnitudes of vocal compensations for –200 cents perturbations relative to the AA (*p* = 0.044) and AG (*p* = 0.011) genotypes. The asterisk indicates significant differences in the magnitudes of vocal responses between the genotype groups. **(D–F)** Pearson correlation analyses revealed significant positive correlations between the magnitudes of vocal compensations and the SDs of the baseline *F*_0_ for the AA (*r* = 0.534, *p* < 0.001) and AG genotypes (*r* = 0.264, *p* = 0.003) but not for the GG genotypes (*r* = 0.107, *p* = 0.500).

**Figures 1A,B** show the grand-averaged *F*_0_ contours in response to -50 and -200 cents perturbations, respectively. The magnitudes of vocal compensations for -200 cents perturbations appear to be influenced by the genotype, showing smaller vocal compensations for the GG genotype relative to the AA and AG genotype. A two-way RM-ANOVA conducted on the magnitudes of vocal responses revealed no significant main effects of stimulus magnitude [*F*(1,130) = 1.678, *p* = 0.198, $\eta_{p}^{2}$ = 0.013] and genotype [*F*(2,130) = 2.222, *p* = 0.112, $\eta_{p}^{2}$ = 0.033]. The interaction between these two variables, however, reached significance [*F*(2,130) = 5.488, *p* = 0.005, $\eta_{p}^{2}$ = 0.078]. Follow-up one-way RM-ANOVAs showed that the magnitudes of vocal responses did not vary as a function of genotype for the -50 cents condition [*F*(2,130) = 0.998, *p* = 0.371, $\eta_{p}^{2}$ = 0.015]. Nevertheless, there was a significant main effect of genotype for the -200 cents condition [*F*(2,130) = 4.484, *p* = 0.013, $\eta_{p}^{2}$ = 0.065]. Bonferroni *post hoc* comparisons showed that the GG genotype was associated with significantly smaller vocal compensations relative to the AA (*p* = 0.044) and AG (*p* = 0.011) genotypes (**Figure 1C**), while the magnitudes of vocal responses did not differ significantly between individuals with AG and AA variants (*p* = 1.000). For the latencies of vocal response, the main effects of stimulus magnitude [*F*(1,130) = 0.065, *p* = 0.799, $\eta_{p}^{2}$ = 0.001] and genotype [*F*(2,130) = 1.288, *p* = 0.279, $\eta_{p}^{2}$ = 0.019] did not reach significance. As well, there was no significant interaction between stimulus magnitude and genotype [*F*(2,130) = 0.268, *p* = 0.765, $\eta_{p}^{2}$ = 0.004].

Pearson correlation analyses were performed to investigate the relationship between the magnitude of vocal compensation and the variability of the baseline voice while hearing normal auditory feedback across the genotypes. This measure is hypothesized to reflect the degree of reliance on auditory feedback in the online monitoring of speech production (Scheerer and Jones, 2012; Huang et al., 2016; Li et al., 2016). The results revealed significant positive correlations between the magnitudes of vocal responses and the SDs of the baseline *F*_0_ for individuals with the AA (*r* = 0.534, *p* < 0.001) (see **Figure 1D**) and AG genotypes (*r* = 0.264, *p* = 0.003) (see **Figure 1E**), indicating that the variability of normal voice *F*_0_ was predicative of the degree of vocal compensation for these two groups. However, this correlation did not reach significance for individuals with the GG genotype (*r* = 0.107, *p* = 0.500) (see **Figure 1F**).

### ERP Findings

**FIGURE 2:**
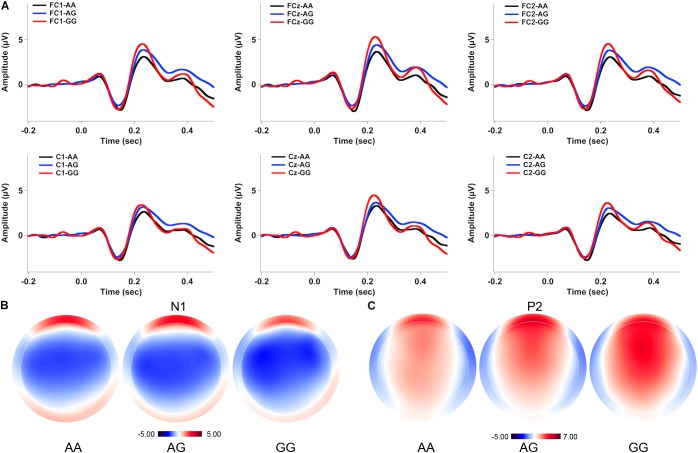
Grand-averaged ERP waveforms **(A)** and topographical distributions of the N1 **(B)** and P2 **(C)** amplitudes in response to –50 cents pitch perturbations across the genotypes. The black, blue, and red solid lines represent cortical ERPs for the AA, AG, and GG genotypes, respectively.

**FIGURE 3:**
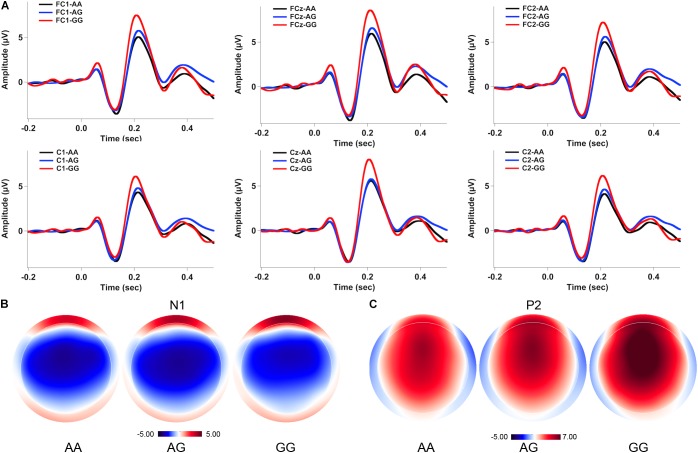
Grand-averaged ERP waveforms **(A)** and topographical distributions of the N1 **(B)** and P2 **(C)** amplitudes in response to –200 cents pitch perturbations across the genotypes. The black, blue, and red solid lines represent cortical ERPs for the AA, AG, and GG genotypes, respectively.

**FIGURE 4:**
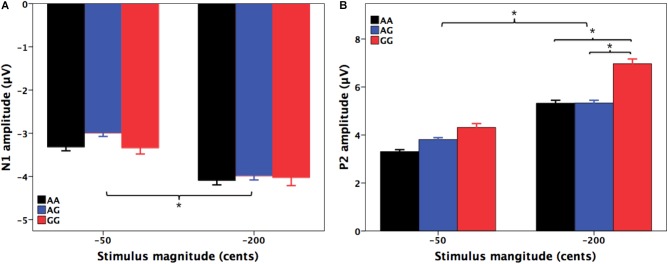
T-bar plots of the amplitudes (mean + SEM) of N1 **(A)** and P2 **(B)** responses to –50 and –200 cents perturbations in auditory feedback for the AA (black), AG (blue), and GG (red) genotypes. The –200 cents condition elicited significantly larger N1 (*p* < 0.001) and P2 (*p* < 0.001) amplitudes than the –50 cents condition. Individuals with the GG genotype produced significantly larger P2 responses to –200 cents pitch perturbations than individuals with the AA (*p* = 0.023) and AG genotypes (*p* = 0.019). The asterisk indicates significant differences in the N1 and P2 amplitudes across the conditions.

**Figures 2, 3** show the grand-averaged ERP waveforms (A) and topographical distributions of the N1 (B) and P2 (C) amplitudes in response to -50 and -200 cents perturbations, respectively. The effects of *FOXP2* genotype on the cortical ERP responses appeared to be more pronounced in the case of the 200 cents condition, showing larger P2 amplitudes for the GG genotype relative to the AA and AG genotypes. A three-way RM-ANOVA revealed that the -200 cents condition elicited significantly larger N1 amplitudes than the -50 cents condition [*F*(1,130) = 39.040, *p* < 0.001, $\eta_{p}^{2}$ = 0.231] (see **Figure 4A**). N1 amplitudes were also found to vary across the electrode sites [*F*(9,1170) = 7.141, *p* < 0.001, $\eta_{p}^{2}$ = 0.052], with larger N1 amplitudes at the frontal electrodes relative to the central electrodes. The main effect of genotype, however, did not reach significance [*F*(1,130) = 0.204, *p* = 0.815, $\eta_{p}^{2}$ = 0.003]. The interactions between any of these variables also did not reach significance (*p* > 0.05).

For the N1 latencies, the -50 cents condition elicited significantly slower N1 responses than the -200 cents condition [*F*(1,130) = 88.820, *p* < 0.001, $\eta_{p}^{2}$ = 0.406]. There was also a significant main effect of electrode site [*F*(9,1170) = 6.068, *p* < 0.001, $\eta_{p}^{2}$ = 0.045]; electrode FC1 was associated with significantly longer N1 latencies than electrodes C1 (*p* = 0.004) and Cz (*p* = 0.017). However, neither the main effect of genotype [*F*(1,130) = 0.100, *p* = 0.905, $\eta_{p}^{2}$ = 0.002] nor the interactions between any of these three variables (*p* > 0.05) reached significance.

A three-way RM-ANOVA conducted on the P2 amplitudes revealed a significant main effect of stimulus magnitude [*F*(1,130) = 194.803, *p* < 0.001, $\eta_{p}^{2}$ = 0.600], showing significantly larger P2 amplitudes for the -200 cents condition relative to the -50 cents condition (see **Figure 4B**). Significantly larger P2 amplitudes at the frontal electrodes relative to the central electrodes led to a significant main effect of electrode site [*F*(9,1170) = 182.553, *p* < 0.001, $\eta_{p}^{2}$ = 0.584]. There was also a significant main effect of genotype [*F*(2,130) = 3.596, *p* = 0.030, $\eta_{p}^{2}$ = 0.052] as well as a significant interaction between stimulus magnitude and genotype [*F*(2,130) = 4.706, *p* = 0.011, $\eta_{p}^{2}$ = 0.068]. Follow-up two-way RM-ANOVAs were conducted across the stimulus magnitudes. For the -50 cents condition, P2 amplitudes did not vary as a function of genotype [*F*(2,130) = 2.627, *p* = 0.076, $\eta_{p}^{2}$ = 0.039], whereas there was a significant main effect of electrode site [*F*(9,1170) = 123.493, *p* < 0.001, $\eta_{p}^{2}$ = 0.487] as well as a significant interaction between genotype and electrode site [*F*(18,1170) = 2.246, *p* = 0.026, $\eta_{p}^{2}$ = 0.033]. Further analyses revealed systematic changes of P2 amplitudes as a function of genotype at electrodes FC1 [*F*(2,130) = 3.202, *p* = 0.044, $\eta_{p}^{2}$ = 0.047], FC2 [*F*(2,130) = 3.505, *p* = 0.033, $\eta_{p}^{2}$= 0.051], FCz [*F*(2,130) = 3.633, *p* = 0.029, $\eta_{p}^{2}$= 0.053], and Cz [*F*(2,130) = 3.056, *p* = 0.050, $\eta_{p}^{2}$= 0.045]. *Post hoc* Bonferroni comparisons revealed that the GG genotype was associated with significantly larger P2 amplitudes relative to the AA genotype (FC1: *p* = 0.048; FC2: *p* = 0.044; FCz: *p* = 0.027; Cz: *p* = 0.046) (see **Figure 2A**). For the -200 cents condition, there was a significant main effect of genotype on the P2 amplitudes [*F*(2,130) = 4.359, *p* = 0.015, $\eta_{p}^{2}$= 0.063]. *Post hoc* Bonferroni comparison revealed that individuals with the GG genotype produced significantly larger P2 amplitudes than individuals with the AA (*p* = 0.023) and AG genotypes (*p* = 0.019) (see **Figures 3, 4B**), whereas P2 amplitudes did not differ significantly between individuals with the AA and AG genotypes (*p* = 1.000). There was also a significant main effect of electrode site [*F*(9,1170) = 186.929, *p* < 0.001, $\eta_{p}^{2}$= 0.590], indicating significantly larger P2 amplitudes at the frontal electrodes relative to the central electrodes. The interaction between electrode site and genotype, however, did not reach significance [*F*(18,1170) = 1.696, *p* = 0.087, $\eta_{p}^{2}$= 0.025].

Regarding the P2 latencies, the main effect of stimulus magnitude [*F*(1,130) = 127.197, *p* < 0.001, $\eta_{p}^{2}$= 0.495] was found to be significant; significantly longer P2 latencies were elicited by the -50 cents condition as compared to the -200 cents condition. The main effect of electrode site [*F*(9,1170) = 2.825, *p* = 0.017, $\eta_{p}^{2}$ = 0.021] also reached significance, indicating that electrode FC1 was associated with significantly longer P2 latencies than electrode C1 (*p* = 0.019). However, P2 latencies did not vary as a function of genotype [*F*(2,130) = 1.541, *p* = 0.218, $\eta_{p}^{2}$= 0.023]. In addition, the interactions between these variables did not reach significance (*p* > 0.05).

### sLORETA Findings

**FIGURE 5:**
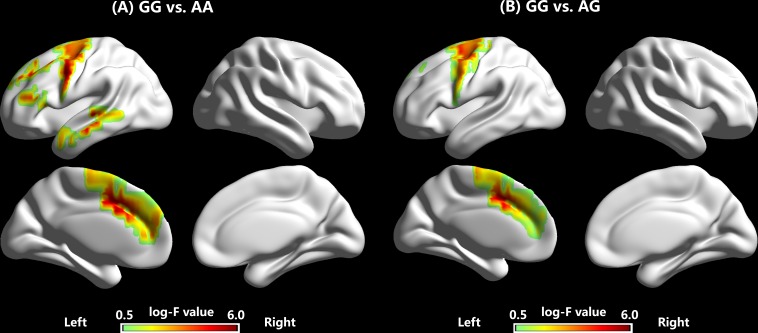
Source reconstructions of the amplitudes of P2 responses to –200 cents pitch perturbations, showing enhanced brain activity in the P2 time windows for individuals with the GG genotype when compared to individuals with the AA **(A)** and AG **(B)** genotypes.

**Table 1** and **Figure 5** show coordinates and corresponding brain regions where individuals with the GG genotype produced significantly larger P2 responses to -200 cents pitch perturbations relative to individuals with the AA and AG genotypes. When compared to the AA genotype, the GG genotype was associated with enhanced P2 responses due to increased brain activity in a left-lateralized neural network, including the left IFG (BA 9, *p* = 0.035), middle frontal gyrus (MFG) (BA 6, *p* = 0.044), precentral gyrus (PrCG) (BA 6, *p* = 0.019; BA9, *p* = 0.043), anterior cingulate cortex (ACC) (BA 32, *p* = 0.017; BA 24, *p* = 0.024), middle temporal gryus (MTG) (BA 21, *p* = 0.041), and insula (BA 13, *p* = 0.007; BA 45, *p* = 0.040) (see **Figure 5A**). Similarly, enhanced P2 responses associated with the GG vs. AG genotype were the result of increased brain activity in the left MFG (BA 6, *p* = 0.012; BA 32, *p* = 0.025), superior frontal gyrus (SFG) (BA 6, *p* = 0.026), and ACC (BA 32, *p* = 0.002; BA 24, *p* = 0.001) (see **Figure 5B**).

**Table 1:** sLORETA *Log*-F statistics for maximum activations obtained from comparisons of the GG genotype with the AA and AG genotypes in the P2 time window (MNI coordinates).

Condition	Brain region	BA	*X*	*Y*	*Z*	Log-*F* value
GG vs. AA	Left IFG	9	-35	5	30	4.375
	Left MFG	6	-45	0	45	4.295
	Left PrCG	6	-35	0	30	4.626
		9	-35	5	40	4.271
	Left ACC	32	-15	5	40	4.652
		24	-15	0	40	4.546
	LFG MTG	6	-60	-25	-10	4.295
	Left insula	13	-30	15	15	4.987
		45	-30	25	5	4.306
GG vs. AG	Left MFG	6	-20	5	55	4.978
		32	-15	10	50	4.675
	Left SFG	6	-20	10	55	4.653
	Left ACC	32	-15	5	40	5.804
		24	-15	0	40	5.831


*IFG, inferior frontal gyrus; MFG, middle frontal gyrus; PrCG, precentral gyrus; ACC, anterior cingulate cortex; MTG, middle temporal gyrus; SFG, superior frontal gyrus.*

## Discussion

The present study investigated the extent to which *FOXP2* variation (SNP rs6980093) was associated with change in the neurobehavioral responses to pitch perturbations during vocal production. When compared to individuals with the GG genotype, individuals with the AA and AG genotypes produced significantly larger vocal compensations for pitch perturbation of -200 cents perturbations that were positively correlated with the variability of their normal voice *F*_0_. Furthermore, individuals with the GG genotype produced significantly larger P2 responses to -200 cents perturbations relative to individuals with the AA and AG genotypes due to left-lateralized increased activity in the SFG, MFG, IFG, PrCG, ACC, MTG, and insula. However, the neurobehavioral responses to -50 cents perturbations did not vary as a function of *FOXP2* genotype. These findings provide the first behavioral and neural evidence that genetic variation in *FOXP2* is associated with sensorimotor integration for vocal pitch regulation.

Our behavioral results of larger vocal compensations associated with the AA and AG genotypes relative to the GG genotype are in line with previous findings of abnormally enhanced vocal compensations produced by individuals with 16p11.2 deletions (Demopoulos et al., 2018). They also parallel findings of incomplete and inaccurate vocal imitation during song learning in young zebra finches with knockdown of *FoxP2* gene (Haesler et al., 2007), and impaired acquisition of motor skills and learning of auditory-motor integration in mice carrying heterozygous *FoxP2* mutations (Groszer et al., 2008; Kurt et al., 2012). More interestingly, individuals with the AA and AG genotypes exhibited significant positive correlations between the degree of vocal compensations and the variability of their normal voice *F*_0_, while no such correlation existed for individuals with the GG genotype. This correlation has been hypothesized to reflect an increased reliance on auditory feedback in sensorimotor control of speech production (Scheerer and Jones, 2012; Chen et al., 2013). In light of the DIVA (directions into velocities of articulators) model (Golfinopoulos et al., 2010), successful control of speech production involves both feedback and feedforward control. Feedforward control allows speakers to correctly produce the speech targets through the internal representations of the motor programs, while feedback control is used to constantly monitor and correct feedback errors for maintaining the accuracy of the internal representations. Decreased reliance on auditory feedback results in an increased reliance on feedforward control and vice versa (Golfinopoulos et al., 2010). Professional singers, who develop a stronger reliance on feedforward control mechanisms, produce significantly smaller vocal compensations than non-musicians (Jones and Keough, 2008) and even are able to successfully ignore large pitch perturbations (Zarate and Zatorre, 2005, 2008). In contrast, significant correlations between vocal variability in patients with PD and TLE and their enhanced vocal compensations for pitch perturbations (Chen et al., 2013; Huang et al., 2016; Li et al., 2016), have been interpreted as an increased reliance on auditory feedback due to impaired feedforward control. This hypothesis has also been used to account for abnormally enhanced vocal compensations produced by individuals with 16p11.2 deletions (Demopoulos et al., 2018). Accordingly, individuals with the AA and AG genotypes may weight auditory feedback more heavily to detect feedback errors from vocal output and thus produce large vocal compensations. By contrast, individuals with the GG genotype may place an increased reliance on feedforward control and tend to “ignore” deviant auditory feedback, thereby producing less of a compensatory response.

Cortically, the GG genotype was associated with significantly larger P2 responses than the AA and AG genotypes, whereas N1 responses did not vary as a function of genotype. These findings may reflect an association between *FOXP2* gene and speech motor control not at the early detection of feedback errors but at the later transformation of auditory feedback into corrective motor commands. Furthermore, enhanced P2 responses associated with the GG genotype received contributions from a left-lateralized network including the SFG, MFG, IFG, ACC, MTG, and insula (see **Figure 5**). Activation of these cortical regions has been identified during auditory-motor control of speech production in previous fMRI (Zarate and Zatorre, 2008; Parkinson et al., 2012; Behroozmand et al., 2015) and ERP studies (Huang et al., 2016; Guo et al., 2017). These results are consistent with the expression of the *FOXP2* gene in the lateral frontal and temporo-parietal cortices (Ferland et al., 2003; Lai et al., 2003) as well as associations between the *FOXP2* gene and activation in the left IFG and PrCG during overt speech production in clinical (Liegeois et al., 2003) and healthy populations (Pinel et al., 2012). Interestingly, a group of musicians with absolute pitch also produced significantly larger P2 responses in the left hemisphere than non-musicians (Behroozmand et al., 2014). Also, professional singers exhibited enhanced brain activity in the ACC, STG, and insula when exposed to vocal pitch errors as compared to non-musicians (Zarate and Zatorre, 2005, 2008). Together with these previous findings, our observation of larger P2 responses in the fronto-temporal regions in individuals with the GG genotype perhaps reflect a more pronounced shift from feedback to feedforward control of vocal production, suggesting that *FOXP2* SNP at rs6980093 may influence the weighting of feedback vs. feedforward control of speech production.

Note that there is a dual-sensory reference frame including both auditory and somatosensory feedback in the DIVA model (Golfinopoulos et al., 2010). Somatosensory feedback provides critical information about speech articulators (Tremblay et al., 2003) and makes significant contributions to speech motor control (Larson et al., 2008; Golfinopoulos et al., 2011; Lametti et al., 2012; Kleber et al., 2013, 2017). The relationship between auditory and somatosensory feedback remains unclear, but there is evidence that these two types of feedback may be in opposition to each other when pitch perturbations are heard. For example, larger vocal compensations for pitch perturbations were elicited by anesthetizing the vocal folds as compared to the pre-anesthetic condition (Larson et al., 2008). Furthermore, Lametti et al. (2012) reported an inverse relationship between reliance on auditory vs. somatosensory feedback: participants who compensated more for somatosensory perturbations compensated less for auditory and vice versa. Therefore, an alternative explanation is that individuals with the GG genotype who exhibited decreased reliance on auditory feedback may weight somatosensory feedback more heavily to attenuate vocal compensations for pitch perturbations when compared to individuals with the AA and AG genotypes, suggesting that the *FOXP2* gene might have an impact on a preferential reliance on sensory feedback.

Our finding that *FOXP2* genetic variation is associated with change in neurobehavioral responses to perceived vocal pitch errors provides an important piece of the puzzle of individual differences in sensorimotor control of speech production. Despite the well-documented large individual variability of vocal compensations (Burnett et al., 1998; Liu P. et al., 2011; Scheerer and Jones, 2012) and cortical ERPs (N1 and P2) (Chen et al., 2012; Li et al., 2013; Behroozmand et al., 2014) elicited by feedback perturbations, much less is known about the causes of these individual differences. Recent evidence has suggested that individual differences in the neurobehavioral processing of vocal pitch errors are related to the participants’ intrinsic brain activity in the fronto-temporal regions (Guo et al., 2016) and the subregional morphology of subcortical structures (Tang et al., 2018). As well, Zhu et al. (2016) found a negative correlation between vocal compensation magnitudes and estradiol levels and an association between increased P2 amplitudes and decreased progesterone levels in young females. Our findings of the relationship between *FOXP2* genetic variant and neurobehavioral responses to pitch perturbations open up a new perspective for linking the genetics to individual variability in speech motor control.

It is worthy noting that the effect of *FOXP2* variation was not observed on the neurobehavioral responses to -50 cents perturbations, which may be related to the differential neural mechanisms that underlie the processing of small and large pitch perturbations in voice auditory feedback. For example, vocalization-induced suppression, as demonstrated by smaller N1 responses to pitch perturbations at vocal onset during active vocalization relative to passive listening, was significantly reduced as the size of the pitch perturbation increased (Behroozmand and Larson, 2011). Cantonese speakers produced smaller vocal and larger P2 responses to -200 and -500 cents perturbations than Mandarin speakers, whereas this group difference did not exist in the case of -50 and -100 cents perturbations (Liu et al., 2010; Chen et al., 2012). Professional singers were more capable of suppressing compensatory vocal responses to 200 cents perturbations (closer to 0 cent) than to 25 cents perturbations with increased activity in the right STG, superior temporal sulcus, left planum temporale and supramarginal gyrus (Zarate and Zatorre, 2008; Zarate et al., 2010), whereas this pattern did not exist in non-musicians (Scheerer et al., 2013). In an analogous way to musicians, individuals with the GG genotype may be better at suppressing vocal compensations for large pitch perturbations than for small pitch perturbations due to their decreased reliance on auditory feedback as compared to individuals with the AA and AG genotypes, leading to decreased vocal compensations and increased P2 responses in the condition of -200 cents perturbations.

Clearly, several inherent limitations of the present study must be acknowledged. First, the sample size obtained for analysis is relatively small, although it is consistent with a number of previous studies linking genetics to speech/language processing or disorders (Zhao et al., 2010; Pinel et al., 2012; Chandrasekaran et al., 2015; Xie et al., 2015). As well, our sample did not have an equal number of female and male across the three genotypes. These confounding factors may lead to low statistical power and high false-discovery rates. Future work should be conducted in a larger sample with a balance of sexes. Second, we examined the association between a single *FOXP2* SNP (rs6980093) and sensorimotor control of speech production, but variability in neuroanatomy was not affected by this SNP (Hoogman et al., 2014). Whether and how this SNP can influence sensorimotor integration without altering brain structure needs to be further investigated. In addition, a comprehensive assessment of auditory processing and cognitive function should be performed in the future studies, since compensatory control of speech production involves many perception and production processes that demand high-level cognitive processing (Liu et al., 2015; Guo et al., 2017). Despite these limitations, our findings offer a starting point for the examination of the mechanisms of speech motor control from a genetic perspective.

## Author Contributions

HL and SZ designed the experiments. SZ, JZ, and ZG performed the experiments and analyzed the data. SZ, JJ, PL, and HL interpreted the results and wrote the manuscript. All authors read and approved the final manuscript.

## Conflict of Interest Statement

The authors declare that the research was conducted in the absence of any commercial or financial relationships that could be construed as a potential conflict of interest.
